# Effects of Substrate Temperature on Optical, Structural, and Surface Properties of Metal–Organic Vapor Phase Epitaxy-Grown MgZnO Films

**DOI:** 10.3390/nano14231957

**Published:** 2024-12-05

**Authors:** Jiamin Liu, Deng Xie, Zhe Chuan Feng, Manika Tun Nafisa, Lingyu Wan, Zhi-Ren Qiu, Dong-Sing Wuu, Chuanwei Zhang, Jeffrey Yiin, Hao-Hsiung Lin, Weijie Lu, Benjamin Klein, Ian T. Ferguson, Shiyuan Liu

**Affiliations:** 1 State Key Laboratory of Intelligent Manufacturing Equipment and Technology, Huazhong University of Science and Technology, Wuhan 430074, China; jiaminliu@hust.edu.cn (J.L.); chuanweizhang@hust.edu.cn (C.Z.); 2 School of Electronic & Electrical Engineering and Physics, Fujian University of Technology, Fuzhou 350000, China; dengx@fjut.edu.cn; 3 Southern Polytechnic College of Engineering and Engineering Technology, Kennesaw University, Marietta, GA 30060, USA; mnafisa@students.kennesaw.edu (M.T.N.); jyiin@kennesaw.edu (J.Y.); bklein8@kennesaw.edu (B.K.); ianf@kennesaw.edu (I.T.F.); 4 Science Exploring Laboratory, Arbour Glenn Drive, Lawrenceville, GA 30043, USA; 5 Center on Nano-Energy Research, Guangxi Key Laboratory for the Relativistic Astrophysics, Institute of Science and Technology for Carbon Peak & Neutrality, School of Physical Science & Technology, Guangxi University, Nanning 530004, China; lyw2017@gxu.edu.cn; 6 State Key Laboratory of Optoelectronic Materials and Technologies, School of Physics, Sun Yat-sen University, Guangzhou 510275, China; stsqzr@mail.sysu.edu.cn; 7 Department of Applied Materials and Optoelectronic Engineering, National Chi Nan University, Puli, Nantou 54561, Taiwan; dsw@ncnu.edu.tw; 8 Department of Electrical Engineering, Graduate Institute of Photonics and Optoelectronics, National Taiwan University, Taipei 10617, Taiwan; hhlin@ntu.edu.tw; 9 Hexagonal Scientific Laboratory, LLC, Dayton, OH 45459, USA; wlu@hsl-mat.com; 10 Optics Valley Laboratory, Wuhan 430074, China

**Keywords:** Mg_0.1_Zn_0.9_O, metal–organic vapor phase epitaxy, X–ray diffraction, spectroscopic ellipsometry, Raman spectroscopy, enharmonic phonon process, synchrotron radiation, near-edge X–ray absorption fine structure, X–ray photoelectron spectroscopy

## Abstract

MgZnO possesses a tunable bandgap and can be prepared at relatively low temperatures, making it suitable for developing optoelectronic devices. Mg*_x_*Zn_1−*x*_O (*x*~0.1) films were grown on sapphire by metal–organic vapor phase epitaxy under different substrate-growth temperatures *T*_s_ of 350–650 °C and studied by multiple characterization technologies like X-ray diffraction (XRD), spectroscopic ellipsometry (SE), Raman scattering, extended X-ray absorption fine structure (EXAFS), and first-principle calculations. The effects of *T*_s_ on the optical, structural, and surface properties of the Mg_0.1_Zn_0.9_O films were studied penetratively. An XRD peak of nearly 35° was produced from Mg_0.1_Zn_0.9_O (0002) diffraction, while a weak peak of ~36.5° indicated MgO phase separation. SE measurements and analysis determined the energy bandgaps in the 3.29–3.91 eV range, obeying a monotonically decreasing law with increasing *T*_s_. The theoretical bandgap of 3.347 eV, consistent with the SE-reported value, demonstrated the reliability of the SE measurement. Temperature-dependent UV-excitation Raman scattering revealed 1LO phonon splitting and temperature dependency. Zn-O and Zn-Zn atomic bonding lengths were deduced from EXAFS. It was revealed that the surface Mg amount increased with the increase in *T*_s_. These comprehensive studies provide valuable references for Mg_0.1_Zn_0.9_O and other advanced materials.

## 1. Introduction

In recent decades, benefiting from the advantages of easily tunable bandgaps larger than 3.2 eV, visible–spectral transparency, considerable exciton-binding energy, good conductivity, non-toxicity, etc., the research and development of ZnO-based materials and structures have attracted significant attention and achieved fruitful results [1,2,3,4,5,6,7,8,9,10,11,12,13,14]. MgZnO ternary alloys, as typical ZnO-based materials, are direct wide-bandgap semiconductors with broad applications in electronic and optoelectronic fields [9,13,15,16]. Ternary Mg*_x_*Zn_1−*x*_O (MZO) alloys with tunable Mg compositions have recently been developed for various high-voltage transparent thin-film transistors and other types of devices [2,9,16]. Meanwhile, by reducing the defect density, the thermal and bias stabilities of MZO alloys can be improved significantly [9,16], which enables much broader applications in novel optoelectronic devices [17]. In these hot and frontier topics involving MZO, tunable bandgap energy between 3.3 and 7.8 eV is realized by changing the Mg or Zn composition in the host ZnO or MgO materials; the corresponding bandgap-based applications have been of the most fundamental concern [16,17,18,19,20,21,22,23,24,25,26,27,28]. For instance, MZO alloys are promising in deep ultraviolet (UV) photodetectors [3], UV light-emitting diodes (LEDs) and transparent thin-film transistors [6], UV thin-film phototransistors [21], n-MZO/p-GaN heterojunction diodes for UV EL applications [24], and bulk acoustic wave (BAW) resonators [25]. Wurtzite structural MZO with *x* < 0.25 is an optimal candidate for applications in optoelectronic devices, such as UV phototransistors, photodetectors, and detectors for UV environmental monitoring [18]. Kumar, P. et al. [2] reported that Mg_0.1_Zn_0.9_O films have room-temperature (RT) ferromagnetism, which is helpful for spintronic applications for energy conversion and storage applications. Also, MZO alloys can be used to construct MZO/CdSeTe/CdTe solar cells with high performance [5] and fabricate MZO/CdSe/CdTe solar cells with improved device characteristics [8]. Usually, MZO films can be prepared via different technologies, such as metal–organic vapor phase epitaxy (MOVPE) [27], molecular-beam epitaxy (MBE) [15,26], electrodeposition [4], atomic layer deposition (ALD) [23], sol–gel and spin-coating methods [24], magnetron sputtering [22], and so on. Substrates used for epitaxy include sapphire [9,22], Si [12,22], and glass [4,5,6,21]. However, how fabrication processes such as substrate-growth temperature affect the surface, structure, and optoelectronic properties of Mg_*x*_Zn_1−*x*_O remains to be investigated.

Several studies have focused on the comprehensive performance characterization and modulation mechanism discovery of Mg*_x_*Zn_1−*x*_O alloys. In these investigations, the variation in the lattice constants of wurtzite Mg*_x_*Zn_1−*x*_O alloys with Mg compositions was characterized using the X-ray diffraction method [6,21,28]. D. C. Tsai et al. [6] used XRD to capture the film crystallinity of crystallite size variations and studied the integral intensity, FWHM, and grain size of MgZnO films as a function of the Al_2_O_3_ content in targets [6]. C. Y. Tsay et al. [21] obtained very narrow grazing incidence XRD peak widths for sol–gel-deposited undoped and Ga-doped Mg_0.2_Zn_0.8_O thin films. Extended X-ray absorption fine structure (EXAFS) spectroscopy, considered a powerful technique, was used to probe the crystalline structures and electronic bonding states of Mg*_x_*Zn_1−*x*_O materials [29,30]. L. Bergman et al. [31] employed ultraviolet photoluminescence and Raman scattering to study Mg*_x_*Zn_1−*x*_O nano-powders with 0 < *x* < 0.26. L. Wang et al. [32] used high-resolution transmission electron microscopy and X-ray diffraction spectroscopy to characterize the growth state of cubic Mg_0.21_Zn_0.79_O/MgO multiple quantum wells. In these studies, the physical properties of Mg*_x_*Zn_1−*x*_O materials were explored under specific substrate temperature conditions, such as 450 °C or 300 °C [27], which motivated us to further investigate the effects of different substrate temperatures.

In the present work, we performed a comprehensive investigation of the optical, structural, and surface properties of a series of Mg_0.1_Zn_0.9_O films on a sapphire substrate prepared by metal–organic vapor phase epitaxy (MOVPE), with different substrate-growth temperatures *T*_s_ of 350, 450, 550, and 650 °C, respectively. These were studied by multiple characterization techniques like high-resolution X-ray diffraction (HR-XRD), spectroscopic ellipsometry (SE), UV (325 nm)-excitation Raman spectroscopy (RS), synchrotron radiation (SR), extended-edge X-ray absorption fine structure (EXAFS) spectroscopy, X-ray photoelectron spectroscopy (XPS), and first-principle calculations. The effects of substrate-growth temperature on the optical, structural, and surface properties of Mg_0.1_Zn_0.9_O films were investigated thoroughly. Detailed studies of Urbach’s tailing energy *E*_U_ [7,21,22] were performed via the SE method.

## 2. Materials and Methods

### 2.1. Preparation and Characterization of Mg_0.1_Zn_0.9_O Films

Experimental Mg_0.1_Zn_0.9_O epitaxial materials were prepared by MOVPE (EMCORE D-180 system, Veeco, New York, NY, USA), with sources of DEZn [Zn(C_2_H_5_)_2_ purity 99.9999%)], CpMg [(C_5_H_5_)_2_Mg purity 99.99%], and oxygen gas [O_2_ (99.999% purity)] plus Ar carry-gas (99.9999% purity). The substrate was set as c-sapphire due to its advantages of low cost, high quality, and suitability for mass production. In order to prepare the high-quality Mg_0.1_Zn_0.9_O, the inner, middle, and outer gas flow rates, as shown in reference [27], were precisely controlled, accompanied by organometallic sources and O_2_ being introduced into the reactor separately and mixing before entering the chamber. The total flows were kept at 2800 sccm, with a pressure of 20 Torr in the reactor, as described in [27]. The Mg amount in Mg_0.1_Zn_0.9_O could be adapted using the control of the CpMg flow rate. In the current work, the sapphire-substrate temperatures *T*_s_ were set as 350 °C, 450 °C, 550 °C, and 650 °C, respectively, enabling a slight modulation of the crystalline quality and the surface Mg amount.

Using XRD observations, the Mg concentration, i.e., *x*(Mg) in the MZO, was determined as about 0.10 for these four MZO films, which were named MZO350, MZO450, MZO550, and MZO650, respectively. Then, the structural, surface, and optical properties of these Mg_0.1_Zn_0.9_O films were evaluated by high-resolution X-ray diffraction (HR-XRD), Raman scattering, and spectroscopic ellipsometry (SE), respectively. Correspondingly, HR-XRD spectra (MRD 5-crystal system, Phillips, USA) were captured and analyzed to reveal the crystallite size, lattice strain, and dislocation density of these samples.

The ellipsometric spectra captured using a Mueller-matrix ellipsometer (ME-L, Wuhan Eoptics Technology Co., Ltd., Wuhan, China) were analyzed to obtain the optical constants, bandgap, thickness, and surface roughness. Ellipsometric parameter spectra (psi *ψ*(λ) and delta Δ(*λ*)) covering a spectrum of 193–1690 nm were captured using the multi-incidence angle probing mode, in which three incidence angles of 65°, 70°, and 75° were used to ensure the convergence of the inverse problem solution involved. A stratified optical model, consisting of an air ambient layer, a roughness layer, an isotropic layer, and the sapphire substrate layer, was used to fit the measured *ψ*(λ) and Δ(*λ*) spectra of the four samples. Following the bottom-up principle, the dielectric functions of the sapphire substrate were first modeled using the Cauchy oscillators, and the dielectric functions of the Mg*_x_*Zn_1−*x*_O layer were represented by parametrized Tauc–Lorentz oscillators. A two-phase effective-medium approximation model was used to describe the dielectric functions of the surface roughness layer on the Mg*_x_*Zn_1−*x*_O layer, and the dielectric functions of the air ambient layer were set to 1. By analyzing the ellipsometric spectra, via the forward optical model built in J. A. Woollam’s CompleteEASE software M2000, the complex refraction index and the thickness could be determined for four Mg*_x_*Zn_1−*x*_O films. The thicknesses of these Mg_0.1_Zn_0.9_O were determined as 87 nm, 82 nm, 74 nm, and 77 nm, accompanying low mean square errors in the fitting of ellipsometric spectra. Variable-temperature Raman scattering spectra were captured using a micro-Raman system (inVia^TM^, Renishaw, UK) combined with a variable-temperature loading module (THMS600, LinKam, Salford, UK). The Raman scattering spectra were obtained under ultraviolet excitation from a He-Cd laser at a wavelength of 325 nm. Further theoretical analyses, based upon the 3- and 4-phonon processes, on the variable-temperature Raman spectra were performed, revealing 1-LO phonon splitting and temperature dependency.

Extended X-ray absorption fine structure (EXAFS) spectroscopy was employed to acquire the atomic bonding lengths of Mg_0.1_Zn_0.9_O films, revealing the crystalline structure and electronic bonding states. The angle-dependent Zn K-edge EXAFS spectra were captured using the fluorescence-mode 17C beamline under the incidence angle range of 15°~90° at the synchrotron radiation research center in Hsinchu. In addition, X-ray photoelectron spectroscopy (XPS) was performed by a ULVAC-PHI PHI 5000, with the excitation source as Al Kα (1486.7 eV) radiation and with the photo-electron take-off angle as 45°. All the spectra were calibrated to a C1s peak at 284.6 eV.

### 2.2. First-Principle Calculations

**Figure 1:**
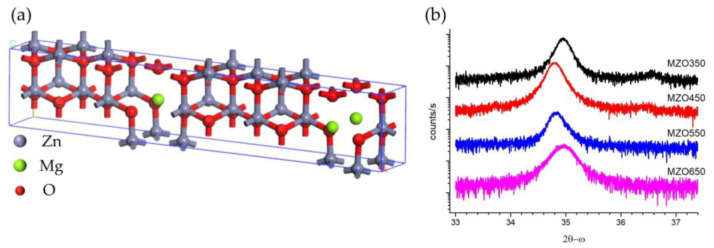
Crystalline structure of Mg_0.1_Zn_0.9_O: (**a**) Lattice structure of Mg_0.1_Zn_0.9_O; (**b**) HR-XRD spectra (0002) of four Mg_0.1_Zn_0.9_O.

Although multi-scale computational studies combined with density functional theory have partially explained the growth mechanism and structural selectivity of many typical wide-bandgap semiconductors [33,34,35], research on the accurate calculation of the bandgap of Mg*_x_*Zn_1−*x*_O and the influence of possible defect states still needs to be improved. All the first-principle calculations based on density functional theory were carried out with the Vienna Ab initio simulation package (VASP v5.4.4), to obtain the band structure, bandgap, and density of state. The exchange-correlation potential was described using the Perdew−Burke−Ernzerhof (PBE) functional of generalized gradient approximation (GGA) based on the projected augmented wave (PAW) pseudopotentials. The original ZnO with a hexagonal wurtzite structure was built by setting the space-group symmetry of P_63_mc and lattice parameters of a = b = 3.249 Å and c = 5.205 Å. After the geometry optimization of the lattice cell, the ZnO supercell 2 × 5 × 1 was modeled in a configuration containing 40 atoms (20 Zn atoms and 20 O atoms). A doping Mg atom (10 at. % concentration) was chosen, as shown in Figure 1a. Then, we used the PBE functional based on the PAW pseudopotential with an energy cutoff of 600 eV and a 5 × 5 × 3 Г-centered k-point mesh, ensuring the reliable optimization of the geometric structure and the approximative calculation of the physical properties. Meanwhile, the GGA + U scheme with appropriate on-site Coulomb interaction U can correct the Zn-3d electronic orbits, which enables the accurate calculation of the bandgap. The reference U perturbation for the Zn-3d orbitals is set to U_Zn3d_ = 12.6 eV. Also, the effects of Mg doping in the ZnO crystallite structure on the electronic and optical properties were evaluated and analyzed. When calculating the projected density of states, a denser 10 × 10 × 6 Г-centered k-point mesh was used. The convergence criteria of force and total energy were 0.01 eV/Å and 10^−6^ eV, respectively.

## 3. Results

### 3.1. High-Resolution X-Ray Diffraction Measurement and Calculation

Figure 1b presents the HR-XRD (0002) scanning results for four MgZnO/sapphire samples, indicating that all MgZnO samples grown on the sapphire substrates show an apparent polarity (0002) orientation. The peak near 35° is from MgZnO (0002) diffraction [15,16], while a weak peak near 36.5° appears for two samples with lower (350 °C and 450 °C) growth temperatures. The cubic ZnO possesses a maximum solid solubility of 35% for hexagonal MgO [31], which is related to the XRD peak near 36.5°. This indicates that MgO phase separation could occur at lower substrate-growth temperatures, due to the incorporation of MgO causing zincblende (111) face diffraction. However, the XRD peaks near 36.5° are much weaker than the XRD peaks near 35°, indicating a shallow magnitude of MgO phase separation in MZO350 and MZO450. It can also be seen that the wurtzite (0002) peak for MZO350 and MZO650 is located at 35.0° of 2*θ,* while those for MZO450 and MZO550 are at 34.7° of 2*θ*. This indicates that the c-axis lattice constants of MZO350 and MZO650 are slightly larger than those of MZO450 and MZO550, which reflects that too-low and too-high substrate-growth temperatures could lead to a larger c-axis lattice constant of the MgZnO film.

The crystal phase and lattice constant of these samples can be evaluated from their XRD data. From the Bragger formula 2*d*sin*θ* = *nλ*, 2*θ* = 35.0° indicates a lattice constant of 5.127 Å, consistent with the literature value 5.206 Å [36]. The XRD peak near 36.5° corresponds to a lattice constant of 4.261 Å, representative of hexagonal MgO in cubic ZnO [31] and indicative of MgO phase separation having occurred at a lower substrate-growth temperature. To analyze the structural properties of four MgZnO samples and compare their crystal qualities, the HR-XRD (0002) peak and FWHM values from Figure 1b were obtained by Gaussian fitting, and those are listed in Table 1. Detailed quantitative calculations were performed to obtain the crystallite size *D*, lattice strain *ε*, and dislocation density *δ* of Mg_0.1_Zn_0.9_O [4,5,6], as shown in Table 1.

The FWHMs for four MgZnO films are located in the range of 0.51°–0.76°, compatible with MgZnO film XRD FWHM values of 0.5°–1.0° from D. C. Tsai et al. [6], implying good crystal quality. Four MgZnO samples have a low threading dislocation density between 3.8 and 8.4 × 10^−3^ nm^−2^. The sample MZO550, grown at the substrate-growth temperature of 550 °C, possesses the largest crystallite size and the lowest lattice strain and dislocation density. This phenomenon might be attributed to the modulating effects of substrate-growth temperature on the MgZnO crystalline state, which is similar to the observations of the annealing temperature effects reported by C.L. Heng et al. [24]. They found that the XRD (0002) peak’s FWHM decreases from 0.391° to 0.157° with the annealing temperature increasing from 600 °C to 900 °C, corresponding to the average size of MgZnO nanocrystals increasing from 21.0 nm to 52.4 nm. It can be seen that their XRD values [21,24] are superior to ours listed in Table 1, which indicates that we still need to further improve the growth of our MgZnO film materials.

### 3.2. Spectroscopic Ellipsometry Measurement and Analysis for Mg_0.1_Zn_0.9_O Films

Spectroscopic ellipsometry (SE) measures the relationships of polarization states (psi *Ψ* and delta *Δ*) for the incidence and reflection of light versus the wavelength from a sample. The roughness, thickness, refractive index *n*, and extinction coefficient *k* of Mg_0.1_Zn_0.9_O films were derived from SE data. SE spectra of Mg_0.1_Zn_0.9_O samples are fitted by using J. A. Woollam Co. CompleteEASE software. In the model, the surface roughness is modeled by a Bruggeman effective medium approximation. The optical constants of sapphire are taken from [37] and kept fixed during the fittings. By adjusting all parameters in the Mg_0.1_Zn_0.9_O layer, the best-fitted SE data for four samples were achieved. The film thickness and surface roughness for all Mg_0.1_Zn_0.9_O samples with minimum errors can be obtained. A comparative measurement of SE and cross-section scanning-electron microscopy (SEM) on the thicknesses of AlN thin layers were performed [38], which confirmed the accuracy of the SE technological determination of the nanometer-scale film thicknesses. Therefore, the thicknesses of our Mg_0.1_Zn_0.9_O films determined by SE and listed in Table 1 are feasible and reliable.

**Figure 2:**
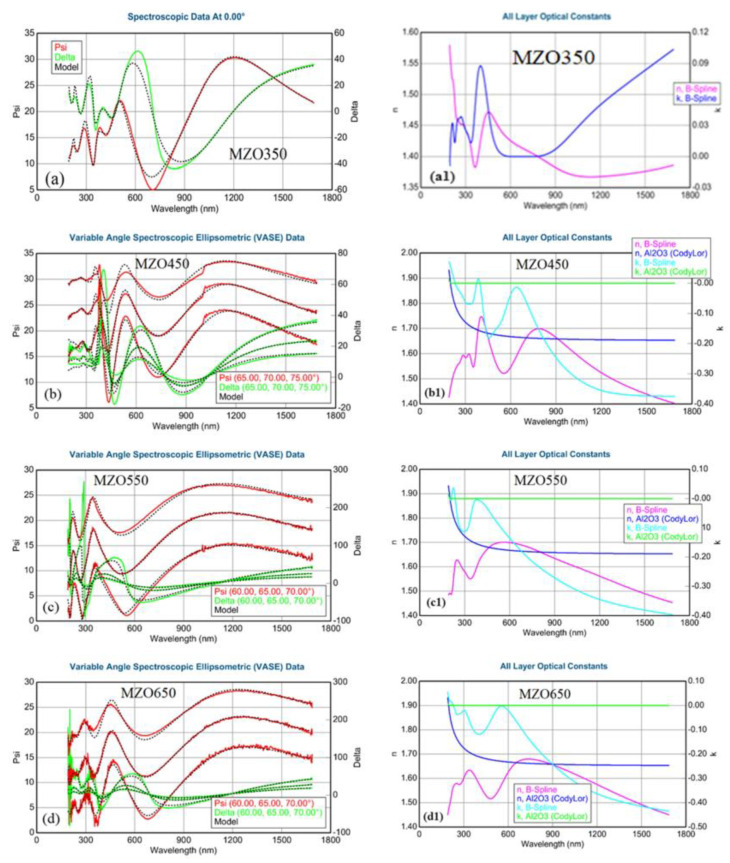
SE psi and delta experimental and fitting *n* and *k* spectra of four MgZnO/sapphire samples. (**a**–**d**) Fitting results of psi and delta for MZO350 (**a**), MZO450 (**b**), MZO550 (**c**), and MZO650 (**d**). (**a1**–**d1**) Complex refractive indexes for MZO350 (**a1**), MZO450 (**b1**), MZO550 (**c1**), and MZO650 (**d1**).

**Figure 3:**
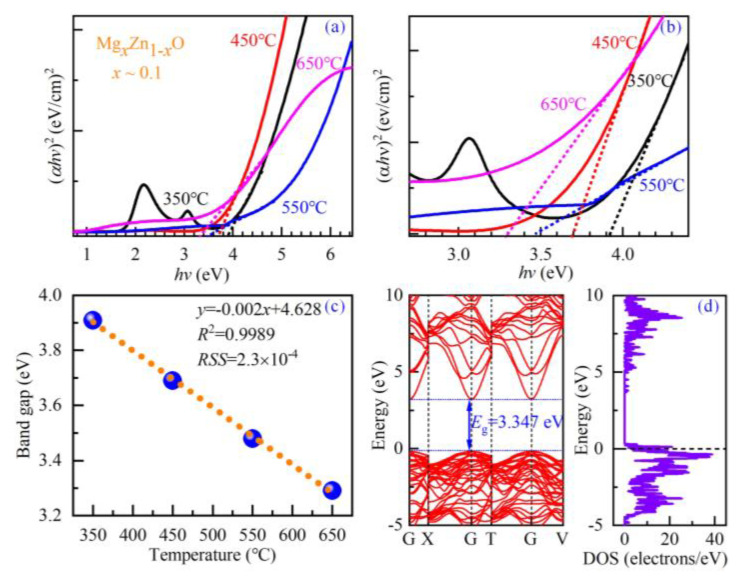
SE-deduced (*αhv*)^2^~*hv* spectra of four MgZnO/sapphire samples of MZO350, MZO450, MZO550, and MZO650: (**a**) in the wide energy range of 0.7–6.5 eV, and (**b**) in 2.7–4.4 eV, showing their bandgap *E*_g_ clearly. (**c**) The substrate-growth temperature dependency of the bandgap energy, and (**d**) the theoretical band structure determined via the first-principle calculations.

Figure 2a–d present the fitting results of *Ψ* and *Δ*, and Figure 2a1–d1 show the optical constants *n* and *k* of the four Mg_0.1_Zn_0.9_O samples. The calculated *Ψ* and *Δ* curves are matched well with the corresponding experimental spectra for all three incidence angles of 60°, 65°, and 70°. The fitted film thicknesses of the four samples are listed in Table 1. In the meanwhile, we can obtain the absorption coefficient *α*(*λ*) = 4π*k* /*λ* from the curve of the extinction coefficient *k* versus the wavelength *λ*. Then, by drawing the curves of (*αhv*)^2^ versus the photon energy *hv* and carrying out a linear interpolation for these four curves [21], the bandgap values could be determined from the intersection points between the interpolation line and the x-axis. The interpolation results are presented in Figure 3, accompanying the bandgaps *E*_g_ of 3.91 eV, 3.69 eV, 3.48 eV, and 3.29 eV (with error bars of about +0.02 eV). The corresponding results are also listed in Table 1. Through the linear fitting of the bandgap *E*_g_ versus the substrate-growth temperature *T*_s_, the temperature dependency of the bandgap can be determined via the following empirical formula,

(1) $$E_{g}=-0.002T_{s}+4.628,$$

where the coefficient of determination *R*^2^ and the residual sum of squares *RSS* are 0.9989 and 2.3 × 10^−4^, implying a satisfactory fitting analysis in Figure 3c. The fitting results show that the substrate-growth temperature has a linear modulation effect on the bandgap of Mg_0.1_Zn_0.9_O, manifested as a monotonically decreasing law. Meanwhile, the energy band structure and the electronic density of state are calculated using first-principle simulations, as shown in Figure 3d. It can be easily found that the valence band maxima and conduction band minimum are located at the gamma point, which implies a direct bandgap of 3.347 eV. The theoretical bandgap is consistent with the measurement value reported by the ellipsometry analysis, indicating the reliability of spectroscopic ellipsometry-based characterizations.

**Figure 4:**
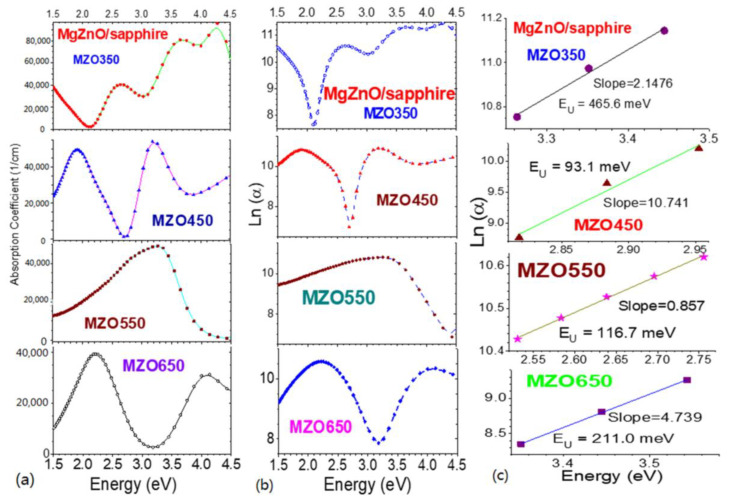
Relationships of absorption coefficient *α* with photon energy *hv* (**a**) and ln[*α*(*hv*)] with photon energy *hv* (**b**), and *E*_U_ determination (**c**), for four MgZnO samples of MZO350-MZO650, respectively. Symbols indicate experimental and calculation data. Connecting lines in the left and middle columns are guides for the eyes.

It can be observed from Figure 3 that an exponential absorption band tail exists below the band edge in these four Mg_0.1_Zn_0.9_O samples, which may result from the structural disorder accompanying electron–phonon coupling [38,39]. This band tail, named the Urbach energy *E*_U_, can be determined by the following analysis of the curves of the absorption coefficient *α* versus the photon energy *hv* [38,39,40],

(2) EU−1=dlnαhvdhv,

Therefore, the Urbach energy *E*_U_ can be acquired from the reciprocal of the slope on the linear part for the curves of the logarithm Ln(*α*) versus the photon energy *hv* [40]. Figure 4 exhibits four Mg_0.1_Zn_0.9_O samples grown at 350–650 °C, showing the relationships of absorption coefficients and their logarithm versus photon energy, i.e., *α*~*hv* and ln[*α*(*hv*)]~*hv* spectra, which finally lead to the determination of *E*_U_. The obtained *E*_U_ are listed into Table 1. The lowest-*T*_s_ sample (MZO350) has a large *E*_U_ of 466 meV, and the highest-*T*_s_ sample (MZO650) has the 2nd-largest *E*_U_ of 211 meV, while the other two samples, with *T*_s_ of 450 °C and 550 °C, possess *E*_U_ values of 93 meV and 117 meV, respectively. These results are consistent with those from D.-C. Tsai et al. [6] and C.-Y. Tsay et al. [21].

When correlating the Urbach tail energy *E*_U_ and the XRD (0002) peak’s FWHM in these four Mg_0.1_Zn_0.9_O samples, it can be easily noted that MZO550 possesses the narrowest XRD (0002) peak’s FWHM, lowest dislocation density, and near-lowest *E*_U_. A close correlation exists between the SE-derived *E*_U_ and the XRD FWHM/dislocation density, which implies that the properties in the area near the band edge are related to crystal quality.

### 3.3. UV (325 nm) Excitation Raman Scattering and Temperature Behavior

**Figure 5:**
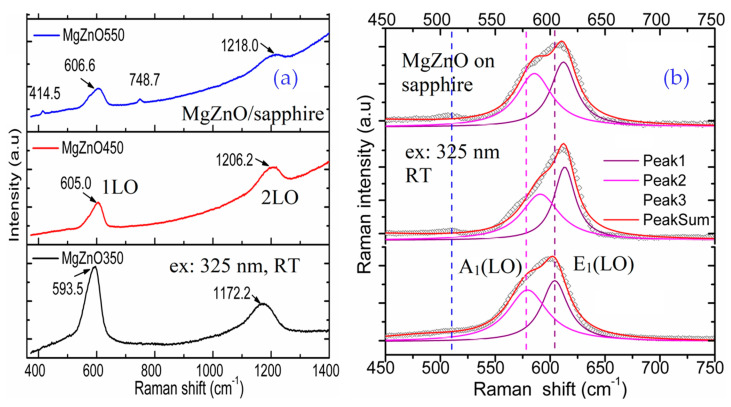
UV 325 nm laser-excited Raman spectra at room temperature of three Mg_0.1_Zn_0.9_O samples for (**a**) 1−LO and 2−LO modes, and (**b**) Gaussian fits on 1−LO with A_1_(LO) and E_1_(LO).

Figure 5 shows 325 nm wavelength laser-excited Raman spectra of four Mg_0.1_Zn_0.9_O samples. Because of the Mg_0.1_Zn_0.9_O samples’ thickness of less than 100 nm, the Raman scattering spectra in the visible band showed robust features from the sapphire substrate [15], overwhelming the Mg_0.1_Zn_0.9_O signals. Meanwhile, the 325 nm wavelength laser-excited Raman measurements overcame these difficulties. The excitation photon energy of 3.815 eV matched the samples’ bandgaps in 3.4–3.9 eV, showing that a resonant enhancement of the Raman longitudinal optical (LO) mode intensities was achieved [31].

Figure 5a presents the 1-LO and 2-LO spectra under the 325 nm wavelength laser excitation. In comparison with the pure ZnO 1-LO at 577 cm^−1^ and 2-LO at 1154 cm^−1^ [31], our Mg_0.1_Zn_0.9_O samples’ 1-LOs shifted to high frequencies of 14–28–30 cm^−1^ and 2-LOs shifted by 19–54–64 cm^−1^, respectively. With the *T*_s_ increase of 350–450–550 °C, the 1-LO and 2-LO frequencies shifted monotonically, and accompanying this, the shift amounts increased. Two dim peaks appeared at 415.5 and 748.7 cm^−1^ in the sample MZO550, caused by the sapphire substrate, which was not found in the other two samples. These appeared because the MZO550 sample had a film thickness of 74 nm, lower than 87 nm for MZO350 and 82 nm for MZO450, from Table 1.

It can be observed that all the 1-LO modes in Figure 5a have obvious asymmetric line shapes, which can be recognized as a mixed A_1_-E_1_ symmetry phonon or a quasi-LO mode [31]. The mixing and broadening are indicative of both inhomogeneous alloy broadening and the presence of defects [31]. Each 1-LO mode can be deconvoluted into two Gaussian peaks, i.e., A_1_(LO) and E_1_(LO) modes, which are shown in Figure 5b. J. Huso et al. previously carried this out for Mg_0.2_Zn_0.8_O nanoalloys [41], where the nanoalloys’ Raman spectrum was well-fitted to the two peaks at 574 cm^−1^ and 601 cm^−1^, with a separation of 27 cm^−1^.

Besides A_1_(LO) and E_1_(LO) modes, there is a weak mode near 510 cm^−1^, indicated by a dashed blue straight line in Figure 5a. Similar weak features near the Raman shift positions were observed by C. Bundesmann et al. [42], when they studied Raman scattering spectroscopy of Mg*_x_*Zn_1−*x*_O films and attributed it to the Mg in ZnO. Accordingly, we also tentatively assign the weak mode at ~510 cm^−1^ to Mg in ZnO.

It can be observed from Figure 5b that from MZO350-MZO450-MZO550, the A_1_(LO) peak frequency varies in V-shape, while the E_1_(LO) peak frequency shows a monotonic blue shift. The split between the A_1_(LO) and E_1_(LO) peaks, ∆*ω* = *ω*[A_1_(LO)] − *ω*[E_1_(LO)], varies with the variation in the substrate-growth temperature. The physical mechanisms underlying the splitting and the A_1_(LO)/E_1_(LO) shifts are still under investigation. K. V. S. Ganesha et al. [43] predicted that the Raman line frequency shift in the LO phonon mode would be related to the oxygen defects, interstitial zinc, and free carriers. Meanwhile, J. Huso et al. [41] pointed out that phonon dynamics due to the order–disorder state of the alloy is a very plausible mechanism. They further demonstrated that possible mechanisms of LO line broadening relevant to the MgZnO alloy include granular morphologies in Mg*_x_*Zn_1−*x*_O films that may introduce structural defects such as grain boundaries and dangling bonds [44], compositional fluctuation that results in inhomogeneous broadening, and crystal anharmonicity. It is indicated that *E*_1_ is the main symmetry component in the quasi-LO mode [31]. Therefore, it can be reasonably inferred that the *E*_1_(LO) mode is influenced by varying *T*_s_ less than the A_1_(LO) mode, which leads to the E_1_(LO) mode frequency alternating less with *T*_s_ than that of the A_1_(LO) mode, as shown in Figure 5b.

**Figure 6:**
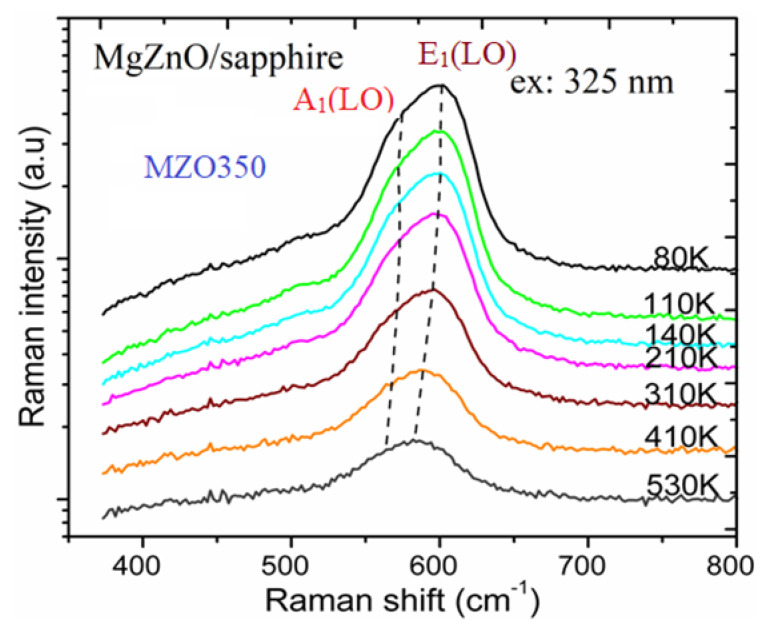
Temperature-dependent Raman spectra of the MZO350 sample between 80 and 530 K.

To further explore the thermodynamic characteristics, we performed variable-temperature Raman measurements and analyses on these three Mg_0.1_Zn_0.9_O samples. Figure 6 exhibits temperature-dependent Raman spectra of the MZO350 sample, measured in 80–530 K. The same experiments were also performed for the other two MgZnO samples, MZO450 and MZO550. The Raman peak near 600 cm^−1^ can be deconvoluted into A_1_(LO) and E_1_(LO) modes, similar to that shown in Figure 5b. In Figure 6, two dashed lines indicate the variations in these two modes. It can be seen that the split between A_1_(LO) and E_1_(LO), ∆*ω* = *ω*[A_1_(LO)] − *ω*[E_1_(LO)], is decreased with the temperature increasing from 80 K to 530 K.

**Figure 7:**
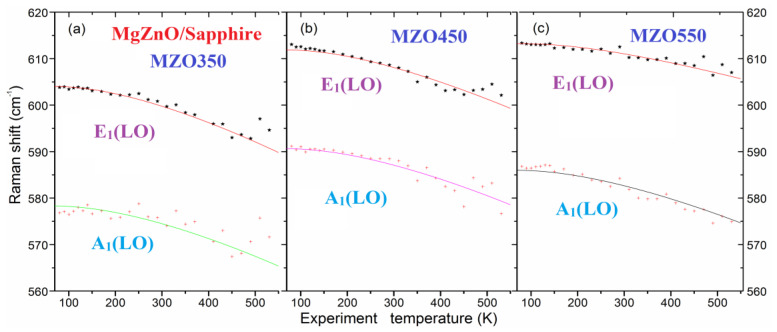
Fitting results of the Raman shift versus *T* of the E_1_(LO) and A_1_(LO) for three Mg_0.1_Zn_0.9_O samples of MZO350 (**a**), MZO450 (**b**), and MO550 (**c**), respectively. Symbols are for experimental data, and fitted results from Equation (6) are represented by solid lines.

C. Bundesmann et al. [42] indicated that for lattice vibrations with A_1_ and E_1_ symmetries, the atoms move parallel and perpendicular to the c-axis, respectively. This means that the A_1_(LO) mode describes the phonon vibration along the c-axis, while the E_1_(LO) mode describes that perpendicular to the c-axis. Our experimental data in Figure 6 reveal that with *T* increasing from 80 K to 530 K, the lattice constant along the c-axis varies less than that perpendicular to the c-axis, i.e., the lattice constant c of Mg_0.1_Zn_0.9_O varies less with *T* than the lattice constants of a and b. Further theoretical exploration was carried out on this anharmonic process. A model involving three- and four-phonon processes was used to fit the temperature-dependent Raman shift and width. The temperature-dependent Raman frequency *ω*(*T*) can be modeled as the following formula [45,46,47],

(3) $$\omega \left(T\right)=\omega_{0}+\omega^{\left(1\right)}\left(T\right)+\omega^{\left(2\right)}\left(T\right),$$

where *ω*_0_ is the phonon frequency at temperature *T* = 0 K, *ω*^(1)^(*T*) is the contribution from linear thermal expansion, and ω^(2)^(*T*) represents the contribution due to phonon coupling. The second term ω^(1)^(*T*) can be denoted as,

(4) $$\omega^{\left(1\right)}\left(T\right)=\omega_{0}\cdot \left\{\exp\left[-\gamma \int_{0}^{T}\left[\alpha_{c}\left(t\right)+2\alpha_{a}\left(t\right)\right]dt\right]-1\right\},$$

where *γ* is the Gruneisen parameter for the phonon mode, which presents the anharmonicity of the crystal lattice, and *α*_c_(t) and *α*_a_(t) are the linear thermal expansion coefficients along the c- and a-axes, respectively. The third term *ω*^(2)^(T) can be expressed as,

(5) $$\omega^{\left(2\right)}\left(T\right)=M_{1}\cdot \left\{1+\sum_{i=1}^{2}\frac{1}{\exp(x_{i})-1}\right\}+M_{2}\left\{1+\sum_{j=1}^{3}\left[\frac{1}{\exp(y_{j})-1}+\frac{1}{{\left[\exp\left(y_{j}\right)-1\right]}^{2}}\right]\right\},$$

where *M*_1_ and *M*_2_ are empirical parameters for fits. The *x_i_* and *y_j_* values satisfy Σ*x_i_* = Σ*y_j_* = *ħω*_0_. The first term of *ω*^(2)^(T) denotes the three-phonon process, and the second describes the four-phonon process. During the data-fitting procedure, we took fixed values of *x* = *x*_1_ = *x*_2_ = *ħω*_0_/2 and *y* = *y*_1_ = *y*_2_ = *ħω*_0_/3. The temperature dependence of the Raman frequency *ω*(*T*) can be simplified as,

(6) $$\begin{array}{l} \omega \left(T\right)=\omega_{0}+\omega^{\left(1\right)}\left(T\right)+\omega^{\left(2\right)}\left(T\right)=\omega_{0}\cdot \exp\left[-3\gamma \int_{0}^{T}\alpha \left(t\right)dt\right]\\ +M_{1}\cdot \left[1+\frac{2}{e^{x}-1}\right]+M_{2}\cdot \left[1+\frac{3}{e^{y}-1}+\frac{3}{{\left(e^{y}-1\right)}^{2}}\right] \end{array},$$

where *ω*_0_, *M*_1_, and *M*_2_ are the parameters for fits. Figure 7 shows the fitting results for the curves of the Raman shift versus *T* of three Mg_0.1_Zn_0.9_O samples, accompanying the fitting parameters listed in Table 2. The results indicate that the E_1_(LO) fitting below 300 K is good but with noticeable error bars at high temperatures, of 400–530 K. Meanwhile, for A_1_(LO), only MZO450 has good fitting results below 300 K, but with noticeable error bars in other cases.

The line width *Γ*, i.e., FWHM, of Raman modes can be deduced from anharmonic interactions. Similar to the interpretation of the temperature-dependent Raman frequency *ω*(*T*), the temperature dependency of the Raman line width *Γ*(*T*) can be given by [45,46,47],

(7) $$\Gamma \left(T\right)=\Gamma_{0}+N_{1}\cdot \left[1+\frac{2}{e^{x}-1}\right]+N_{2}\cdot \left\{1+\frac{3}{e^{y}-1}+\frac{3}{{\left(e^{y}-1\right)}^{2}}\right\},$$

where *Γ*_0_ is the mode line width at 0 K due to impurity and defect scattering. Quantities *N*_1_ and *N*_2_ are fitting parameters. The second and third terms of *Γ*(*T*) represent three- and four-phonon processes, respectively. Equations (3)–(7) have been successfully used in analyzing the temperature-dependent Raman behaviors of GaN [45], ZnO [46], and 4H-SiC [47].

**Figure 8:**
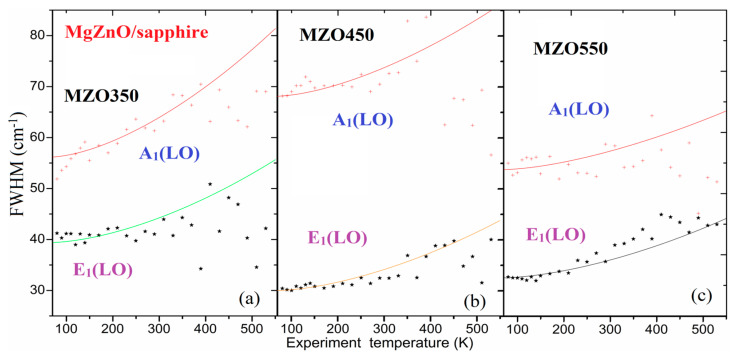
Fitting results of the Raman shift versus *T* of A_1_(LO) and E_1_(LO) for three MZO350 (**a**), MZO450 (**b**), and MO550 samples (**c**), respectively. Symbols correspond to the experimental Raman data, and solid lines represent the fitted results.

Figure 8 presents fitting results obtained by applying Equation (7) to the temperature-dependent width *Γ*(*T*) in 60–530 K for the three samples (MZO350, MZO450, and MZO550), accompanying the fitting parameters listed in Table 3. This shows that the error bars in Figure 8 are large, and only rough trends with *T* are obtained. The trend of rough matching between the measurement and fitting data implies that the temperature dependency of the Raman shift could mainly be due to lattice expansion from the thermal dynamics process and the anharmonic process from three- and four-phonon coupling.

**Figure 9:**
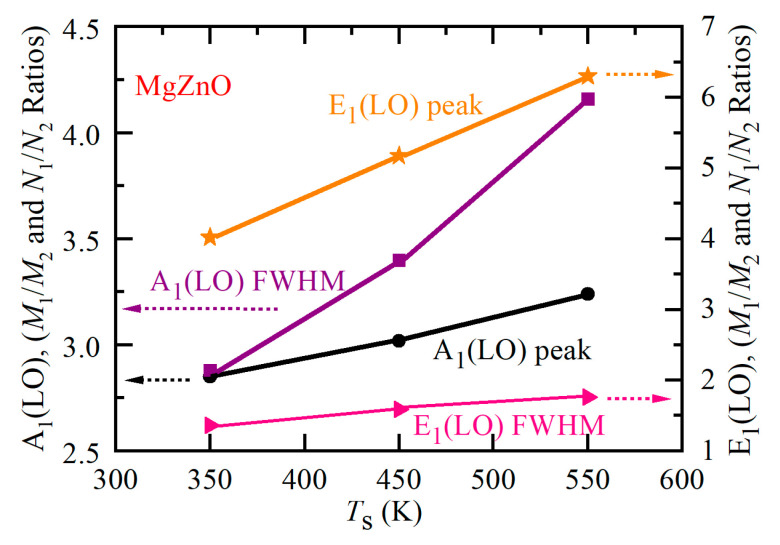
*M*_1_/*M*_2_ and *N*_1_/*N*_2_ versus *T*_s_ of the Mg_0.1_Zn_0.9_O samples.

Table 2 and Table 3 show that with the substrate-growth temperature *T*_s_ increasing from 350 °C to 550 °C, *M*_1_/*M*_2_ and *N*_1_/*N*_2_ ratios are decreased, indicating the decrease in the four-phonon process. Figure 9 further presents the variations in these two ratios versus substrate-growth temperature.

Based upon the above fittings in Table 3 on FWHM and the obtained *Г*_0_, for A_1_(LO) and E_1_(LO) of the three Mg_0.1_Zn_0.9_O samples, we calculated the phonon lifetimes. The phonon lifetime *τ* of a phonon mode obeys the energy–time uncertainty relation [48,49],

(8) ΔE=ℏ/τ=2πcГ,

where ∆*E* is the phonon line width, and *ħ* = 5.3 × 10^(−12)^ cm^−1^·s. *τ* = *ħ*/Δ*E* = 5.3/*Г*_0_ (ps) can be calculated, and the obtained values are listed in Table 4. It can be seen that the phonon lifetimes of the A_1_(LO) mode (in 0.08–0.11 ps) are shorter than those of the E_1_(LO) mode (in 0.15–0.2 ps) for all three Mg_0.1_Zn_0.9_O samples. Among A_1_(LO) phonons, MZO450 has a phonon lifetime shorter than MZO350 and MZO550, while for E_1_(LO) with the reverse trend, MZO450 possesses a phonon lifetime longer than the other two.

In the literature search, we did not find the phonon lifetimes of ternary MgZnO alloys, so we attempted to make comparisons with those from binary ZnO and AlN semiconductors. R. Cuscó et al. [48] studied the *T*-dependence of Raman scattering in ZnO and obtained the phonon lifetimes at 300 K of A_1_(LO) and E_1_(LO) modes, which were 0.54 ps and 0.45 ps, respectively, longer than values of our MgZnO samples. B. P. Swain measured C-doped ZnO with longer phonon lifetimes [49]. The phonon lifetimes of A_1_(LO) and E_1_(LO) modes (in about 0.1–0.2 ps) from our Mg_0.1_Zn_0.9_O samples were shorter than those of binary ZnO from above [48] in the literature and AlN thin films (0.4–0.8 ps) studied by us previously [50], but still at the same 0.1 ps order level. In the future, we will try to further study the phonon lifetime of Mg*_x_*Zn_1−*x*_O alloys with a wide range of *x*(Mg). C. Aku-Leh et al. [51] and M. Mil-lot et al. [52] performed *T*-dependent Raman studies on ZnO materials and obtained the *T*-dependences of ZnO phonon lifetimes, which offer good references. In addition, for cases of other types of oxides, such as rutile tin dioxide (SnO_2_), T. Lan et al. [53] performed variable-temperature (VT, 83–873 K) Raman studies and first-principles calculations, to assess the kinematics of three- and four-phonon processes, which generate Raman peak widths and shifts. L.K. Gaur et al. [54] investigated the phonon anharmonicity and microstructural changes of laser-power-dependent Raman spectra in Co-doped SnO2 nanoparticles, revealed a large Raman mode broadening and shift towards the lower wavenumber side, and presented an explanation based on the kinematics of three-phonon processes.

### 3.4. Extended X-Ray Fine Structure (EXAFS) Investigation

**Figure 10:**
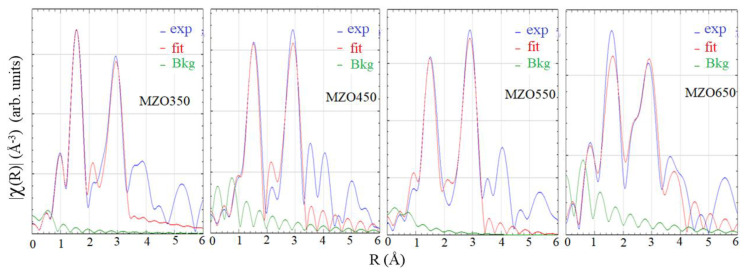
The Zn *K*-edge EXAFS and the fitting spectra of the four Mg_0.1_Zn_0.9_O samples.

Extended X-ray fine structure (EXAFS) spectroscopy is a powerful technique to investigate the crystal electronic structure, and we employed it to study the configuration of Mg and Zn atoms. We had previously performed EXAFS studies for a set of Mg*_x_*Zn_1−*x*_O (0 < *x* < 0.15) layers deposited on sapphire by MOCVD [29,30]. EXAFS spectra of the Zn *K*-edge from our four Mg_0.1_Zn_0.9_O samples were captured using the same technique. Subsequently, normalized and background-subtracted Zn *K*-edge fine structure spectra *K*^2^·*χ*(*K*) were obtained for our samples. Utilizing the EXAFS fitting program IFEFFIT, the Fourier-transformed fitting results of the real-space spectra were produced, as shown in Figure 10, accompanying the fitting parameters listed in Table 5.

In Table 5, *R*_in_(Zn-O) represents the bonding length between the nearest Zn and O atoms in the c-plane, and *R*_out_(Zn-O) represents the bonding length between the nearest Zn and O atoms along the c-axis direction. The four nearest Zn atoms form a tetrahedral structure. *R*_in_(Zn-Zn) represents the bonding length between the nearest Zn and Zn atoms along the a-axis direction. *R*_out_(Zn-Zn) represents the bonding length between the nearest Zn and Zn atoms out of the c-plane. Because *x*(Mg) values are near 0.1, the two peaks in the EXAF spectra are caused mainly by Zn-O and Zn-Zn bonding. From Table 5, the nearest bonding length *R*_in_(Zn-O) is between 1.939 and 1.987 Å, while the *R*_in_(Zn-O) of MZO350 and MZO650 is longer than that of MZO450 and MZO550. Also, as the substrate-growth temperature *T*_s_ increases, the in-plane Zn-Zn bonding length *R*_in_(Zn-Zn) increases, accompanying the out-of-plane Zn-Zn bonding length *R*_in_(Zn-Zn) decreasing. This trend matches well with XRD data analysis and can be explained as follows. With the substrate-growth temperature *T*_s_ rising, Mg integrates into the hexagonal ZnO structure, leading to the increase of the surface *x*(Mg) amount and the alteration of the Zn-Zn bonding length.

## 4. Conclusions

Mg*_x_*Zn_1−*x*_O (*x*~0.1) thin films were grown on sapphire with different substrate growth temperature Ts of 350 °C, 450 °C, 550 °C, and 650 °C, respectively, by MOCVD and investigated via multiple techniques. The effects of the substrate-growth temperature *T*_s_ on the optical, structural, and surface properties of Mg_0.1_Zn_0.9_O thin films were studied penetratively. HR-XRD data confirmed the Mg_0.1_Zn_0.9_O (0002) diffraction, indicated that MgO phase separation occurred at a low *T*_s_, and revealed that too-low and too-high *T*_s_ lead to the expansion of the c-axis lattice constant of the Mg_0.1_Zn_0.9_O film. Calculations indicated that the sample with *T*_s_ = 550 °C had the largest crystallite size and the lowest lattice strain and dislocation density. SE measurements and analyses determined the Mg_0.1_Zn_0.9_O film thicknesses, allowed us to chart (*α·hv*)^2^ versus *hv* plots, and determined their bandgap *E*_g_ in the 3.29–3.91 eV range. The obtained *α* versus *hv* and Ln(*α*) versus *hv* relationships determined the Urbach energy *E*_U_, between 93 and 466 meV.

UV-excitation Raman spectra exhibited the Mg_0.1_Zn_0.9_O asymmetric 1-LO modes deconvoluted into the A_1_(LO) and E_1_(LO) modes. Variable-temperature (VT) Raman analyses revealed that their splitting, ∆*ω* = *ω*[A_1_(LO)] − *ω*[E_1_(LO)], decreased with *T* from 80 K to 530 K. A theoretical model with three- and four-phonon processes was used to fit the *T*-dependent Raman shift and width. The matching trend implies that the Raman shift versus *T* dependences occurs mainly due to lattice expansion from the thermal dynamics process and the anharmonic process involving three- and four-phonon coupling. The A_1_(LO) mode has a phonon lifetime *τ* longer than that of the E_1_(LO) mode. MZO450 has a *τ*[A_1_(LO)] longer than those of MZO350 and MZO550, while its *τ*[E_1_(LO)] is shorter than the other two.

The Zn *K*-edge extended X-ray fine structure (EXAFS) investigation deduced both in-plane Zn-O and out-of-plane Zn-Zn atomic bonding lengths. The nearest bonding length *R*_in_(Zn-O) is between 1.939 and 1.987 Å, while the *R*_in_(Zn-O) of MZO350 and MZO650 are longer than those of MZO450 and MZO550. As the *T*_s_ increases, the in-plane Zn-Zn bonding length *R*_in_(Zn-Zn) increases, while the out-of-plane Zn-Zn bonding length *R*_out_(Zn-Zn) decreases. This trend matched the XRD results. As the *T*_s_ rises, more Mg integrates into the hexagonal ZnO structure, and the addition of surface Mg content results in the alteration of the Zn-Zn (Mg) bonding length.

Our results revealed that *T*_s_ = 550 °C is the optimal *T*_s_ for MOCVD growth of Mg_0.1_Zn_0.9_O on sapphire substrate, and they provide valuable references for Mg_0.1_Zn_0.9_O and other advanced materials.

## Figures and Tables

**Table 1:** XRD (0002) 2*θ* peak and FWHM; calculated values of crystallite size, lattice strain, and dislocation density; SE-deduced film thickness, bandgap *E*_g_, and Urbach energy *E*_U_ of four MgZnO (MZO) films.

Sample No.	MZO350	MZO450	MZO550	MZO650
Substrate-growth temperature (*T*_s_)	350 °C	450 °C	550 °C	650 °C
Peak 2*θ* (0002) (°)	35.00	34.80	34.82	34.96
FWHM 2*θ* (0002) (°)	0.526	0.710	0.513	0.763
Crystallite size *D* (nm)	15.84	11.73	16.24	10.92
Lattice strain *ε* (×10^−3^)	2.19	2.96	2.14	3.18
Dislocation density *δ* = 1/D^2^ (×10^−3^ nm^−2^)	3.99	7.27	3.79	8.39
Thickness (nm) from SE	87	82	74	77
*E*_g_ (eV) from SE	3.91	3.69	3.48	3.29
*E*_U_ (meV) from SE	466	93	116	211

**Table 2:** VT Raman peak fitting parameters.

Parameters		MZO350	MZO450	MZO550
A_1_(LO)	*ω*_0_	580.2	592.4	587.6
	*M*_1_	−1.48	−1.42	−1.33
	*M*_2_	−0.52	−0.47	−0.41
	*M*_1_/*M*_2_	2.85	3.02	3.24
E_1_(LO)	*ω*_0_	606.8	614.4	614.1
	*M*_1_	−2.35	−2.24	−0.89
	*M*_2_	−0.55	−0.42	−0.14
	*M*_1_/*M*_2_	4.27	5.33	6.36

**Table 3:** VT Raman peak fitting parameters.

Parameters		MZO350	MZO450	MZO550
A_1_(LO)	*Г*_0_	50.1	63.3	50.4
	*N*_1_	4.41	3.64	2.62
	*N*_2_	1.53	1.07	0.63
	*N*_1_/*N*_2_	2.88	3.40	4.16
E_1_(LO)	*Г*_0_	35.9	26.8	29.7
	*N*_1_	2.21	2.08	1.87
	*N*_2_	1.21	1.02	0.85
	*N*_1_/*N*_2_	1.83	2.04	2.20

**Table 4:** The phonon lifetime of A_1_(LO) and E_1_(LO) Raman modes of samples.

Parameters		MZO350	MZO450	MZO550
A_1_(LO)	*Г*_0_ (cm^−1^)	50.1	63.3	50.4
	*τ* (ps)	0.106	0.084	0.105
E_1_(LO)	*Г*_0_ (cm^−1^)	35.9	26.8	29.7
	*τ* (ps)	0.148	0.198	0.178

**Table 5:** Fitted result from the Zn *K*-edge EXAFS spectra for the four Mg_0.1_Zn_0.9_O samples.

Sample No.	*R*_in_(Zn-O) (Å)	*R*_out_(Zn-O) (Å)	*R*_in_(Zn-Zn) (Å)	*R*_out_(Zn-Zn) (Å)
MZO350	1.9472	1.7649	3.1521	3.3027
MZO450	1.9394	1.7557	3.1544	3.2968
MZO550	1.9632	1.8052	3.2776	3.1530
MZO650	1.9870	1.7621	3.3171	3.1395

## Data Availability

The data presented in this study are available on request from the corresponding author.
